# Climate is changing, are European bats too? A multispecies analysis of trends in body size

**DOI:** 10.1002/ece3.10872

**Published:** 2024-02-07

**Authors:** Danilo Russo, Gareth Jones, Adriano Martinoli, Damiano G. Preatoni, Martina Spada, Andrea Pereswiet‐Soltan, Luca Cistrone

**Affiliations:** ^1^ Laboratory of Animal Ecology and Evolution (AnEcoEvo), Dipartimento di Agraria Università degli Studi di Napoli Federico II Portici Italy; ^2^ School of Biological Sciences University of Bristol Bristol UK; ^3^ Unità di Analisi e Gestione delle Risorse Ambientali, Guido Tosi Research Group, Dipartimento di Scienze Teoriche ed Applicate Università degli Studi dell'Insubria Varese Italy; ^4^ Istituto Oikos, Via Crescenzago Milano Italy; ^5^ Institute of Systematics and Evolution of Animals, Polish Academy of Sciences Warsaw Poland

**Keywords:** altitude, Bergmann's rule, Chiroptera, climate change, latitude

## Abstract

Animal size, a trait sensitive to spatial and temporal variables, is a key element in ecological and evolutionary dynamics. In the context of climate change, there is evidence that some bat species are increasing their body size via phenotypic responses to higher temperatures at maternity roosts. To test the generality of this response, we conducted a >20‐year study examining body size changes in 15 bat species in Italy, analysing data from 4393 individual bats captured since 1995. In addition to examining the temporal effect, we considered the potential influence of sexual dimorphism and, where relevant, included latitude and altitude as potential drivers of body size change. Contrary to initial predictions of a widespread increase in size, our findings challenge this assumption, revealing a nuanced interplay of factors contributing to the complexity of bat body size dynamics. Specifically, only three species (*Myotis daubentonii*, *Nyctalus leisleri*, and *Pipistrellus pygmaeus*) out of the 15 exhibited a discernible increase in body size over the studied period, prompting a reassessment of bats as reliable indicators of climate change based on alterations in body size. Our investigation into influencing factors highlighted the significance of temperature‐related variables, with latitude and altitude emerging as crucial drivers. In some cases, this mirrored patterns consistent with Bergmann's rule, revealing larger bats recorded at progressively higher latitudes (*Plecotus auritus*, *Myotis mystacinus*, and *Miniopterus schreibersii*) or altitudes (*Pipistrellus kuhlii*). We also observed a clear sexual dimorphism effect in most species, with females consistently larger than males. The observed increase in size over time in three species suggests the occurrence of phenotypic plasticity, raising questions about potential long‐term selective pressures on larger individuals. The unresolved question of whether temperature‐related changes in body size reflect microevolutionary processes or phenotypic plastic responses adds further complexity to our understanding of body size patterns in bats over time and space.

## Graphical abstract

This study spanning over 20 years and involving 15 bat species in Italy challenges the expectation of a widespread increase in body size in response to climate change. Contrary to predictions, only three out of the 15 species exhibited a noticeable size increase, prompting a revaluation of bats as reliable indicators of climate change based on body size alterations. We identify temperature‐related variables, sexual dimorphism, latitude, and altitude as crucial factors influencing bat body size dynamics, revealing a nuanced interplay that adds complexity to our understanding of how bats respond to environmental changes over time and space.

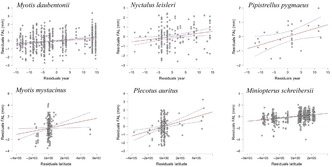

## 1 INTRODUCTION

Climate change is undeniably affecting many facets of animal biology, encompassing morphology (Ryding et al., 2021; Sheridan & Bickford, 2011), biomechanics (Domenici & Seebacher, 2020), physiology (Bradshaw & Holzapfel, 2010; Mitchell et al., 2018; Pörtner & Farrell, 2008), ecology (Hill et al., 2021; Kubelka et al., 2022; Scheffers et al., 2016), and behaviour (Buchholz et al., 2019). While some species may thrive in a warmer environment (Roeder et al., 2021; Sandoval‐Castillo et al., 2020; Schneider et al., 2022; Tayleur et al., 2016), accumulating evidence suggests that climate change often yields adverse consequences, leading to a loss of biodiversity (Habibullah et al., 2022). A range of adverse reactions with impacts on animal populations has been recorded, including, among others, increased mortality due to thermal stress or dehydration (Welbergen et al., 2008), alterations in reproductive patterns and development (Milligan et al., 2009), prey–predator phenological mismatches (Damien & Tougeron, 2019), shifts in pathogen distributions (Gallana et al., 2013), changes in interspecific competition (Elmhagen et al., 2017), and exacerbated conflicts with humans (Abrahms et al., 2023).

The initial discernible “fingerprints” of climate change in animals encompass alterations in species' geographic ranges (Smeraldo et al., 2021; Thomas, 2010), shifts in phenological events such as changes in migration timing and reproductive patterns (Parmesan & Yohe, 2003), and, intriguingly, changes in body size (Gardner et al., 2011). The prevailing trend toward decreased body size in response to climate change was primarily interpreted as an adaptation to enhance heat dissipation in warmer conditions (Sheridan & Bickford, 2011). However, it is crucial to acknowledge that coping with climate‐induced episodes such as heatwaves might lead to an opposite response, as larger body sizes can help conserve water due to a reduced surface‐to‐volume ratio, aiding in avoiding dehydration (Gardner et al., 2011). Adding to the complexity, changes in body size may also be driven by various ecological factors, including increased food availability or alterations in land use, making it challenging to isolate purely climate‐driven patterns (Yom‐Tov & Geffen, 2011).

Body size is an outcome of a multifaceted interplay of ecological and evolutionary pressures, encompassing factors such as phylogeny (Ashton, 2004; Naisbit et al., 2011), sexual selection (Janicke & Fromonteil, 2021), growth conditions (Toïgo et al., 2006), body heat regulation, and water management (Gardner et al., 2011). Identifying factors that influence body size patterns is therefore especially challenging, and studying these patterns across multiple species can provide valuable insights, particularly when patterns might be less informative at the individual species level.

Bats, a diverse mammal order comprising over 1460 species and distributed worldwide excluding polar regions (Simmons & Cirranello, 2023), provide invaluable ecosystem services (Kunz et al., 2011), including insect control (Tuneu‐Corral et al., 2023), pollination (Macgregor & Scott‐Brown, 2020), and seed dispersal (van Toor et al., 2019), with substantial implications for human well‐being. However, bats are highly vulnerable due to their intrinsic k‐selected life history and their susceptibility to environmental changes such as habitat loss, pesticide exposure, wind turbines, light pollution, invasive species, pathogen spread, and climate change (Frick et al., 2020; O'Shea et al., 2016).

As facultative heterotherms (Dzal et al., 2021), bats are directly influenced by temperature in various aspects of their lives. Temperature significantly affects the seasonal selection of roosts, with hibernacula typically cooler than reproductive sites (Altringham, 2011). In the former, torpor is favoured by lower temperatures (e.g., Geiser, 2004), while in the latter, warmer roosts help mitigate the energy costs of maintaining a constant body temperature during pregnancy and lactation (e.g., Russo et al., 2017). Climate change can disrupt these roosting patterns in multiple ways, potentially exposing some bat species to the adverse effects of heatwaves and turning warm roosts into ecological traps associated with high mortality (Crawford & O'Keefe, 2021; Flaquer et al., 2014; Salinas‐Ramos et al., 2023).

Increasing body sizes in bats over time, linked to climate change, have been documented in several species (Mundinger et al., 2021; Salinas‐Ramos, Agnelli, Bosso, Ancillotto, Russo, 2021; Stapelfeldt et al., 2023). An extensive body of research on Bechstein's bats (*Myotis bechsteinii*) in Germany has demonstrated that female body size has increased in response to rising temperatures (Mundinger et al., 2021). Such an increase in size may be driven by higher roost temperatures, which accelerate embryo development and lead to larger individuals (Mundinger, van Schaik, et al., 2023). This increase is associated with higher mortality, at least in Bechstein's bats (Mundinger et al., 2021), yet not in other species (Stapelfeldt et al., 2023), possibly due to greater food requirements imposed by a relatively large body size, which might not be fully sustained in areas of suboptimal food availability. Bats compensate for this higher mortality by increasing their reproductive rate (Mundinger et al., 2022). Furthermore, the growth in body size is a phenotypically plastic response that can be experimentally induced by heating bat boxes (Mundinger, van Schaik, et al., 2023; Mundinger, Wolf, et al., 2023).

Given these observations, it is reasonable to question the extent to which body size increases in response to climate change are widespread among bats. However, conducting multispecies studies is challenging due to the limited availability of long‐term temporal data generated by longitudinal studies, often constrained by funding limitations and the growing demands for research output in modern academia (Festa et al., 2023).

In this study, we focused on 15 bat species in Italy, for which body size measurements spanning a sufficiently long period (20+ years) were available. Italy has experienced a discernible pattern of rising temperatures associated with climate change (Bocchiola & Diolaiuti, 2010; Brunetti et al., 2006; Polemio & Casarano, 2008). We hypothesize that body size would change over time and predict an increase similar to the findings in the German studies on Bechstein's bats (Mundinger et al. 2021, 2022; Mundinger, van Schaik, et al., 2023; Mundinger, Wolf, et al., 2023), and partly Natterer's bats *Myotis nattereri* (Stapelfeldt et al., 2023). We also considered other potential influencing factors, such as latitude, altitude, and sex. Latitude and altitude are predicted to influence body size following the classical “Bergmann's law” pattern (Bergmann, 1847), favouring larger individuals at higher latitudes or altitudes, i.e., in colder climates, to conserve body heat (e.g., Blackburn & Hawkins, 2004; Teplitsky & Millien, 2014). However, the “rule” is seen as controversial, and even for the many cases that confirm it (Meiri & Dayan, 2003), heat retention is not always a satisfactory explanation (Ashton et al., 2000). Despite the exceptions, such influences cannot be disregarded, particularly when samples cover significant latitudinal or altitudinal ranges. Additionally, sexual dimorphism, where female bats are larger than males, occurs in many species (e.g., Myers, 1978; O'Mara et al., 2016) and needs to be controlled to prevent biases in the sample's sex ratio, which could falsely attribute body size patterns to other factors, including time. Moreover, the larger females may show different trends from those seen in males.

## 2 MATERIALS AND METHODS

### 2.1 Fieldwork

Bats have been captured under licence in the years 1995–2023 across the entire Italian territory, in a range of environmental situations (foraging and drinking sites, commuting routes, and near roost entrances). Techniques employed to catch bats included the use of 6, 9, and 12 m mistnets and harp traps, and in some cases, the Sussex Autobat, an acoustic lure that increases capture success when mistnetting bats in forests (Hill & Greenaway, 2005).

Captures were generally conducted for 3–5 h after sunset. Within this context, we took measurements on each captured bat, encompassing assessments of body mass, employing a digital scale, with a sensitivity to the nearest 0.1 g, and forearm length (FAL), using a calliper with a precision to the nearest 0.1 mm. Bats were identified to the species level according to Dietz and Kiefer (2016), and cryptic species were identified and used for further analysis only when their identification was fully reliable based on molecular analysis, unique diagnostic features (e.g. penis shape in whiskered bats), or echolocation (end frequency and frequency of maximum energy recorded on release from *Pipistrellus pipistrellus* and *Pipistrellus pygmaeus*; e.g., Russo & Jones, 2002).

Sexual differentiation was ascertained through the examination of genital morphology (Racey, 1988). Juveniles were identified by the presence of cartilaginous epiphyseal plates in their finger bones and the presence of more slender finger joints (Anthony, 1988). To discern the reproductive status of adult bats, we categorized males into reproductive or nonreproductive groups based on the observation of enlarged testicles or swollen epididymides. For female bats, classification encompassed pregnant, lactating, postlactating, or nonreproductive states. Lactation status among female bats was confirmed by the gentle manipulation of the mammary region, eliciting extrusion of milk (Racey, 1988).

### 2.2 Data analysis

For our aims, we used FAL as a reliable indicator of body size (e.g., Mundinger et al., 2021; Salinas‐Ramos, Agnelli, Bosso, Ancillotto, Russo, 2021; Salinas‐Ramos, Agnelli, Bosso, Ancillotto, Sánchez‐Cordero et al., 2021) and limited our analysis to (fully grown) adult bats. Since preliminary data exploration showed that small latitudinal differences did not affect FAL, latitude was only added to the models when its range for a species' dataset was sufficiently large (>2°). Latitude, altitude, and year were treated as continuous variables, while “sex” was used as a categorical term. “Latitude” was used as a metric value within the WGS84 system, and “altitude” was expressed in m above sea level (a.s.l.). To investigate the impact of these factors on FAL, we used Multiple Linear Regression with Categorical Predictors using Dummy Coding, in combination with a best subset selection approach. This technique involves evaluating all possible combinations of predictor variables and selecting the subset that results in the optimal model, based on specific criteria. These criteria encompass maximizing the number of significant predictors and achieving strong goodness of fit, as indicated by the adjusted *R*‐squared value, while also prioritizing model simplicity to guard against overfitting. In some instances, nonsignificant factors were included when their presence in the model notably increased the adjusted R‐squared values. In essence, we prioritize models that, all other criteria being equal, minimize the number of model terms. Significance was set at *p* < .05 for all tests. The statistical analyses were performed using JASP 0.17.0.2.1 (JASP Team, 2023) for all tests.

## 3 RESULTS

Overall, we analysed data from 4393 bats from 15 species captured since 1995 and covering spatial and temporal ranges (Table 1; Table S1).

**TABLE 1:** Sample size, sex ratio, forearm length (FAL) values, temporal (years) and altitude ranges, and minimum and maximum latitudes for 15 bat species in Italy.

Species	Sample size		Mean FAL (mm)		Years	Alt. Range (m a.s.l.)	Latitude	
	Females	Males	Females	Males			Min	Max
*Rhinolophus euryale*	78	37	48.0 ± 1.0	47.4 ± 0.9	2002–21	195–334	41.28	42.16
*Myotis bechsteinii*	17	15	41.9 ± 1.1	41.2 ± 0.7	2005–23	29–1412	40.37	42.23
*Myotis capaccinii*	47	15	41.9 ± 0.9	40.9 ± 1.2	2005–23	195–876	41.28	42.15
*Myotis crypticus*	44	79	38.7 ± 1.2	38.7 ± 0.9	2000–22	3–1740	38.23	43.74
*Myotis daubentonii*	255	364	37.6 ± 1.0	36.8 ± 1.0	1995–2023	2–1558	41.27	46.29
*Myotis emarginatus*	193	1107	40.3 ± 1.1	38.5 ± 1.0	2000–23	29–1290	39.74	45.44
*Myotis mystacinus*	55	174	34.6 ± 1.6	34.2 ± 1.4	2000–23	95–1553	38.50	46.49
*Plecotus auritus*	93	90	39.5 ± 1.0	38.8 ± 1.1	2000–22	870–1691	38.50	46.47
*Barbastella barbastellus*	192	55	40.1 ± 1.2	38.9 ± 0.8	2000–22	95–1403	40.47	42.55
*Nyctalus leisleri*	44	156	44.0 ± 1.1	43.2 ± 1.1	1995–2023	211–1610	38.50	46.48
*Hypsugo savii*	185	190	34.1 ± 1.1	32.9 ± 1.0	2000–23	250–1553	40.31	42.22
*Pipistrellus kuhlii*	61	37	34.0 ± 1.1	33.3 ± 0.9	2000–23	29–1324	40.81	42.17
*Pipistrellus pipistrellus*	82	71	31.8 ± 0.7	30.9 ± 0.9	2000–23	21–1527	40.37	46.30
*Pipistrellus pygmaeus*	10	17	32.1 ± 0.5	30.4 ± 1.0	2000–23	876–1234	41.74	41.82
*Miniopterus schreibersii*	286	344	46.0 ± 0.8	45.9 ± 0.8	2002–22	20–1283	40.47	45.69

Here, we present only the most robust subset of multiple linear regression models with categorical predictors predominantly comprising significant factors (Table 2). The full ANOVA and coefficient tables for the best models are provided in Tables S2–S16, while adjusted R‐squared values, overall levels of significance, and significant predictors for all term combinations are displayed in Table S17.

**TABLE 2:** Multiple regression models with best subset selection for forearm length (mm) in 15 bat species in Italy, featuring adjusted *R* ^2^ values, model significance (*p*‐values), and significant factors.

Species	Best subset	Adj*R* ^2^	Model significance	Significant factors
*Rhinolophus euryale*	Sex	.065	0.004	Sex
*Myotis bechsteinii*	Sex	.107	0.038	Sex
*Myotis capaccinii*	Altitude, Sex	.172	0.001	Sex
*Myotis crypticus*	–	−.012	0.657	–
*Myotis daubentonii*	Year, Sex	.178	<0.001	Year, Sex
*Myotis emarginatus*	Latitude, Sex	.295	<0.001	Sex
*Myotis mystacinus*	Latitude, Altitude, Sex	.026	0.031	Latitude, Sex
*Plecotus auritus*	Latitude, Altitude, Sex	.183	0.031	Latitude, Sex
*Barbastella barbastellus*	Altitude, Sex	.296	<0.001	Altitude, Sex
*Nyctalus leisleri*	Year, Sex	.111	<0.001	Year, Sex
*Hypsugo savii*	Sex	.250	<0.001	Sex
*Pipistrellus kuhlii*	Altitude, Sex	.248	<0.001	Altitude, Sex
*Pipistrellus pipistrellus*	Sex	.235	<0.001	Sex
*Pipistrellus pygmaeus*	Year, Altitude, Sex	.569	<0.001	Year, Sex
*Miniopterus schreibersii*	Year, Latitude, Sex	.112	<0.001	Latitude, Sex

**FIGURE 1:**
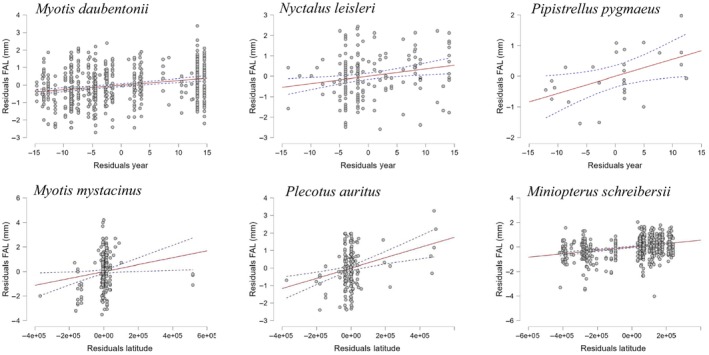
Partial regression plots from a Multiple Linear Regression with Categorical Predictors using Dummy Coding drawn for forearm length (FAL, in mm) vs. year of record (top row) and latitude (bottom row) for six bat species in Italy. Only species for which significant relationships were found are depicted.

Notably, only 20% of the species we considered (*n* = 3) displayed a significant impact of the year of capture on body size: in *M. daubentonii*, *N. leisleri*, and *P. pygmaeus*, body size exhibited an increase in FAL trend over time (Figure 1).

The effects of latitude were only tested for the seven species whose records spanned across a sufficiently large latitude range, i.e., *Myotis daubentonii*, *Myotis emarginatus*, *Myotis mystacinus*, *Nyctalus leisleri*, *Plecotus auritus*, *P. pipistrellus*, and *Miniopterus schreibersii*. Latitude demonstrated a consistent influence on body size for three species, following the classic “Bergmann” pattern, with bats being larger at higher latitudes. This pattern was evident in *P. auritus*, *M. mystacinus*, and *M. schreibersii* (Figure 1). However, in *M. mystacinus*, latitude explained only a minimal amount of sample variability, as evidenced by the small adjusted R‐squared value.

**FIGURE 2:**
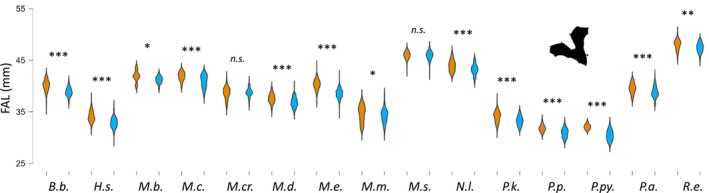
Violin plots of forearm length (FAL) in 15 bat species recorded in Italy, categorized by sex (orange: females; blue: males). Intersexual differences were explored through univariate tests (details are given in Table S17). Levels of significance are expressed as follows: n.s., not significant; *<.05; **<.005; ***<.0001. In all cases, where significant differences were found, females were larger than males. Species abbreviations are as follows: B.b.: *Barbastella barbastellus*; H.s.: *Hypsugo savii*; M.b.: *Myotis bechsteinii*; M.c.: *M. capaccinii*; M.cr.: *M. crypticus*; M.d.: *M. daubentonii*; M.e.: *M. emarginatus*; M.m.: *M. mystacinus*; M.s.: *Miniopterus schreibersii*; N.l.: *Nyctalus leisleri*; P.k.: *Pipistrellus kuhlii*; P.p.: *P. pipistrellus*; P.py.: *P. pygmaeus*; P.a.: *Plecotus auritus*; R.e.: *Rhinolophus euryale*. Sample sizes are shown in Table 1.

Across all species, except for *Myotis crypticus*, sex had a significant influence on the model, with males being smaller than females (Table 1). Univariate *t*‐tests or Mann–Whitney tests confirmed this difference for all species except for *M. crypticus* and *M. schreibersii* (Figure 2; Table S18).

**FIGURE 3:**
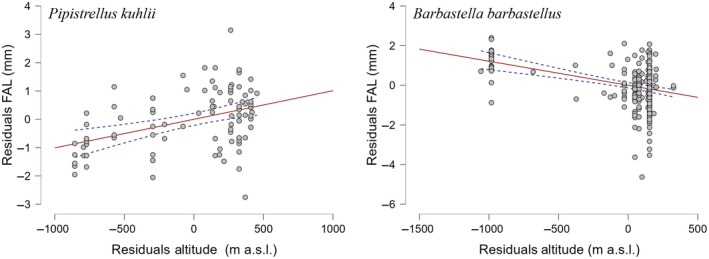
Partial regression plots from a Multiple Linear Regression with Categorical Predictors using Dummy Coding drawn for forearm length (FAL, in mm) vs. altitude for two bat species observed in Italy. Only species for which significant relationships were found are depicted.

After controlling for sexual dimorphism, altitude significantly influenced body size in *Pipistrellus kuhlii* and *Barbastella barbastellus*, however in opposite directions. Bats exhibited larger sizes at higher altitudes in the former, and smaller sizes at higher altitudes in barbastelles (Figure 3).

In the *Hypsugo savii* models that did not include sex as a categorical term, altitude showed a significant effect. However, this was an artefact associated with the fact that males were more frequently captured at high elevations than females, and the former were smaller than the latter. A Mann–Whitney test conducted on altitudes vs. the sex of the bat revealed that median altitudes were greater for captured males compared to females (*U* = 6999.5, *p* < .001). A similar situation was found in *P. pygmaeus*, in which case females were caught more frequently at high altitudes than males, and as females are larger, this simulated an effect of altitude that vanished once sex was added to the models. Again, a Mann–Whitney test conducted on altitudes vs. the sex of the bat revealed that altitudes differed between sexes, this time being higher for females (*U* = 156.0, *p* < .001).

## 4 DISCUSSION

### 4.1 Temporal trends in body size

Our investigation reveals that, among the 15 species under scrutiny, only three exhibited a discernible augmentation in body size over the observed temporal span. This trend was previously documented in Italy for the greater horseshoe bat, *Rhinolophus ferrumequinum*, for which it extended over a century due to the dataset's longevity (Salinas‐Ramos, Agnelli, Bosso, Ancillotto, Russo, 2021). Notably, within the species we considered, this trend was most conspicuous in *Pipistrellus pygmaeus*, as evidenced by a relatively high R‐squared value in multiple regression analysis. Nevertheless, we acknowledge that the limited sample size available for *P. pygmaeus* might have introduced an element of overfitting into our analysis. However, the conspicuous temporal trend for an increase in body size of a riparian bat specialist (*P. pygmaeus*) is also recorded in another riparian species, *Myotis daubentonii*, suggesting potential parallels in the underlying environmental factors influencing this temporal increase.

Additionally, our findings affirm the absence of any temporal shift in the body size of *Pipistrellus kuhlii*, corroborating earlier research spanning 133 years, which focused on specimens from mainland Italy housed in museum collections (Tomassini et al., 2014). Notably, Tomassini et al. (2014) detected an enlargement solely in skull size, commencing shortly after World War II. The authors of that study posited a plausible link between the proliferation of artificial nocturnal lighting during that period in Italy and the associated increased presence of tympanate moths attracted to streetlamps, which, in turn, impaired their antipredatory responses due to the influence of artificial light. Larger skulls would be advantageous to capture and handle moths, bigger than the usual dipteran prey pursued by this bat species in dark habitats (Tomassini et al., 2014). However, we underscore that our present study exclusively examined FAL, leaving open the possibility that other unmonitored morphometric changes occurred within some of the studied species, evading our notice. Climate‐change influences on animal morphology besides overall body size effects have been recorded for several taxa (Anderson et al., 2019; Babin‐Fenske et al., 2008; MacLean et al., 2019; Onley et al., 2020; Wereszczuk et al., 2023). In cave‐dwelling bats such as *M. schreibersii*, the absence of a significant increase in body size over time might be attributed to the colonies' deeper location within karst underground sites, where the effects of climate change on temperature are less pronounced (Mammola et al., 2019).

Any increase in body size over time, as suggested by our findings, is therefore by no means a universal phenomenon. Nevertheless, several limitations of our study merit consideration. Firstly, it is conceivable that such an effect may be contingent upon specific populations. For instance, an upsurge in body size observed in *Myotis nattereri* in Germany manifested solely in a northern population, while a southern population that experienced a warmer climate exhibited no such pattern (Stapelfeldt et al., 2023). Such population‐level distinctions likely account for why the Bechstein's bats included in our study failed to show any temporal alterations in size, in contrast to those examined in Germany (Mundinger et al. 2021, 2022; Mundinger, van Schaik, et al., 2023; Mundinger, Wolf, et al., 2023). Conversely, all size increments identified within Italy (*M. daubentonii*, *N. leisleri*, and *P. pygmaeus*, this study; and *R. ferrumequinum*, Salinas‐Ramos, Agnelli, Bosso, Ancillotto, Russo, 2021) were measured in individuals aggregated from several populations. Notably, the temporal body size increase that we identified in *M. daubentonii* remained conspicuous in a population situated in central Italy whose females also shifted upwards their altitudinal limit (Russo et al., 2024). The compelling evidence indicating that the body size augmentation is instigated by increasingly warm nursery roosts, which favour pregnancy and lactation, ultimately resulting in larger offspring via a phenotypically plastic process (Mundinger, Wolf, et al., 2023), underscores the pivotal role of local factors, including roost structure and microclimate, in either facilitating or impeding the size increase. Alternative explanations to support increasing body size patterns over time in the scientific literature are rare. The FAL increase recorded in *Hipposideros armiger* in China over 65 years was explained as the result of selective pressures acting on body size, enabling the bats to cover longer distances to find the progressively rarer foraging sites (Yue et al., 2020).

The increase in body size we recorded in migratory bat species (*N. leisleri*, and, possibly, *Pipistrellus pygmaeus*; Lindecke et al., 2019) is intriguing. It might be expected that nonmigratory species would be more likely to exhibit morphological responses to climate change than migratory species. Partial migration is likely to occur in migratory bats such as *N. leisleri*, so the individuals we measured may have been given birth and exposed to different temperature trends in different reproductive quarters. Even if for this reason our samples included individuals born in different regions, an increasing trend in body size is still conceivable if temperatures have risen over time in their respective birth regions, especially if the proportion of individuals of different geographic regions remained about constant over time.

### 4.2 Bergmann's rule effects: Latitude and altitude

Our investigation revealed limited effects of latitude on bat body size. However, it is important to note that the available datasets covered a sufficiently large latitudinal range for only 47% of the species under consideration. Therefore, we cannot discount the possibility that latitude may also influence body size in other species for which our dataset was geographically restricted. Among the three species for which a latitude effect was detected, there was a consistent pattern of increasing body size at higher latitudes, conforming to Bergmann's rule. It is worth mentioning that in the case of *Myotis mystacinus*, the observed effect was relatively weak, as indicated by the small adj‐R‐squared value. Additionally, another species, *Rhinolophus hipposideros*, exhibited an increase in body size at higher latitudes in Italy. This observation was based on museum specimens collected across most of Italy over a time exceeding a century (Salinas‐Ramos, Agnelli, Bosso, Ancillotto, Sánchez‐Cordero et al., 2021).

Regarding the response to altitude, which was also anticipated to correlate with body size, we observed such effects in only two species: *Barbastella barbastellus* and *Pipistrellus kuhlii*. However, these effects exhibited opposing directions. In the instance of *Pipistrellus kuhlii*, there was a correlation between larger sizes and higher altitudes, in alignment with Bergmann's rule. A parallel trend emerged in a different pipistrelle species, *P. pipistrellus* in France, where FAL exhibited a negative correlation with temperature (Penone et al., 2018). However, the same species did not exhibit a response to temperature in our current study. Conversely, barbastelles displayed larger body sizes at lower altitudes. These findings corroborate findings by Ancillotto et al. (2015), who observed that barbastelle bats roosting in clay badlands at lower altitudes (106–468 m a.s.l.) exhibited larger body sizes than bats roosting in trees at altitudes exceeding 1200 m a.s.l. The differences in size are most plausibly attributed to phenotypically plastic responses induced by the higher temperatures in the clay badland roosts (Mundinger, van Schaik, et al., 2023; Mundinger, Wolf, et al., 2023). In contrast, bats roosting in dead trees at higher altitudes are exposed to the cold spells typical of mountainous regions, which likely limit offspring development due to reduced thermal insulation (Russo et al., 2017).

Overall, our findings indicate that altitudinal effects on body size are not universally consistent, and their direction is likely influenced by the roosting ecology of the species and the specific microclimatic conditions within their nursery roosts.

The limited support for “Bergmann's rule” that we found may be partly attributed to the relatively narrow altitude and latitude ranges within our study area. Expanding our analysis to encompass the entire European territory could potentially yield different results. Notably, in a study involving 20 bat species covering a substantial portion of North American territory, Alston et al. (2023) observed a latitudinal gradient in body size that aligns with Bergmann's rule. This gradient was characterized by a negative correlation between body mass and mean annual temperature, providing support for the heat conservation hypothesis underlying Bergmann's rule. It is important to acknowledge that body mass in bats is highly variable, influenced by factors such as food availability and individual body condition. Therefore, we opted to employ FAL as a more robust proxy for body size, as has been done in other studies (e.g., Mundinger et al., 2021, 2022; Mundinger, van Schaik, et al., 2023; Mundinger, Wolf, et al., 2023; Salinas‐Ramos, Agnelli, Bosso, Ancillotto, Russo, 2021; Salinas‐Ramos, Agnelli, Bosso, Ancillotto, Sánchez‐Cordero et al., 2021; Stapelfeldt et al., 2023; Tomassini et al., 2014).

### 4.3 Intersexual differences in body size

Our study underscores the statistical significance of body size differences between sexes in most bat species, particularly within two out of the three bat families examined. Notably, *M. schreibersii* from the Miniopteridae family exhibited no discernible intersexual difference in forearm length (FAL).

Traditionally, bats have been regarded as characterized by limited sexual size dimorphism (Lu et al., 2014). However, it is important to recognize that various other morphological, physiological, and behavioural traits exhibit significant disparities between the sexes (Muñoz‐Romo et al., 2021). Although the body size difference we observed may be subtle, with females marginally but significantly larger than males, it is a prevalent phenomenon, particularly evident within families such as vespertilionids (Myers, 1978). This disparity in size has been linked to the physiological necessity of a greater wing loading required to carry the embryo during pregnancy (Myers, 1978; Stevens et al., 2013). Furthermore, in numerous bat species, females are known to carry their offspring clinging to their ventral fur when departing from roosts, particularly during roost switching events (e.g., Russo et al., 2005).

## 5 CONCLUSIONS

Our principal aim was to investigate whether a discernible increase in body size over time, potentially indicative of climate change effects, is evident across various bat species. Contrary to our prediction, however, our examination revealed that only three out of the 15 species under scrutiny showed this specific pattern. Considering these findings, it appears that while bats exhibit promising characteristics as potential indicators of climate change, alterations in their body size may not offer a reliable metric (Russo et al., 2021). One possible challenge lies in establishing the appropriate geographic scale necessary to elucidate responses, which may be specific to populations and can become diluted when individuals from disparate populations exhibiting distinct trends are analysed collectively without accounting for their origin.

Our study has also unveiled a complex interplay of factors influencing body size in bats. Temperature‐related variables, such as latitude and altitude, have emerged as significant drivers in some cases, with the manifestation of Bergmann's‐like patterns suggesting the involvement of natural selection. These spatial dynamics underscore the importance of geographic variations in shaping body size. Furthermore, we propose that the increase in size over time is primarily driven by phenotypic plasticity, as exemplified by the studies done on Bechstein's bats in Germany (Mundinger, van Schaik, et al., 2023; Mundinger, Wolf, et al., 2023). The question of whether this will result in adverse long‐term selective pressures on larger individuals, leading to a shorter lifespan, as observed in Bechstein's bats but not in Natterer's bats (Stapelfeldt et al., 2023), is a subject of debate and warrants longitudinal studies focused on the population level.

The question of whether temperature‐related changes in body size, following Bergmann's rule patterns, primarily stem from microevolution favouring larger body sizes for enhanced heat retention in colder climates or heat dissipation in hotter climates (Salewski et al., 2010) or if they instead reflect a phenotypically plastic response (Teplitsky et al., 2008) remains unresolved. In either scenario, conflicting factors may come into play, as bats inhabiting warmer climates may produce larger offspring while still needing smaller body sizes for more efficient heat dissipation. Additional selective pressures countering a phenotypically plastic increase in body size may arise from the progressively reduced availability of food, a likely scenario in many regions and ecosystems around the world affected by the global decline in insect populations driven by pesticides and changes in land use (Goulson, 2019; Wagner, 2020).

In summary, our study not only highlights the limited occurrence of body size increase over time among bat species but also elucidates the intricate interplay of multiple factors. These factors encompass species‐ or population‐level tendencies in phenotypic responses, species‐specific environmental preferences, geographic variations in roosting site availability, and the potential influence of other limiting factors, such as food resources. In essence, the observed patterns present a mosaic of contrasting pressures that collectively contribute to the complexity of bat body size dynamics, providing insights into the multifaceted nature of ecological and evolutionary processes.

## AUTHOR CONTRIBUTIONS

**Danilo Russo:** Conceptualization (lead); data curation (supporting); formal analysis (lead); funding acquisition (lead); investigation (lead); methodology (lead); supervision (lead); writing – original draft (lead); writing – review and editing (lead). **Gareth Jones:** Investigation (supporting); writing – original draft (supporting); writing – review and editing (supporting). **Adriano Martinoli:** Data curation (supporting); investigation (supporting); writing – original draft (supporting); writing – review and editing (supporting). **Damiano G. Preatoni:** Data curation (supporting); investigation (supporting); writing – original draft (supporting); writing – review and editing (supporting). **Martina Spada:** Data curation (supporting); investigation (supporting); writing – original draft (supporting); writing – review and editing (supporting). **Andrea Pereswiet‐Soltan:** Data curation (supporting); investigation (supporting); writing – original draft (supporting); writing – review and editing (supporting). **Luca Cistrone:** Conceptualization (supporting); data curation (lead); investigation (supporting); methodology (supporting); writing – original draft (supporting); writing – review and editing (supporting).

## Supporting information


Table S1

Tables S2–S16

Table S17

Table S18

## Data Availability

All data used for the preparation of this article are provided in Table S1.
