# Supplementary material for: Climate is changing, are European bats too? A multispecies analysis of trends in body size
Source: Ecol Evol. 2024 Feb 7;14(2):e10872. doi: 10.1002/ece3.10872 (PMC10850807; doi:10.1002/ece3.10872)
Supplement: Supplementary file 2 — Tables S2–S16 [file ECE3-14-e10872-s001.docx]

**Supplementary materials – Tables S2-S16**

Table S2. Analysis of Variance (ANOVA) and Coefficient Tables for the Best Subset Multivariate Regression Model of ***Rhinolophus euryale***.

| ANOVA | | | | | | | | | | | | | | | | |  |
| --- | --- | --- | --- | --- | --- | --- | --- | --- | --- | --- | --- | --- | --- | --- | --- | --- | --- |
| Model | |  | | | Sum of Squares | | | df | | Mean Square | | F | | P | | |  |
| H₁ |  | Regression | |  | 8.479 |  | | 1 |  | 8.479 |  | 8.861 |  | 0.004 | |  |  |
|  |  | Residual | |  | 108.122 |  | | 113 |  | 0.957 |  |  |  |  |  |  |  |
|  |  | Total | |  | 116.601 |  | | 114 |  |  |  |  |  |  |  |  |  |
|  | | | | | | | | | | | | | | | | |  |
|  | | | | | | | | | | | | | | | | |  |
| Coefficients | | | | | | | | | | | | | | | | | |
| Model | |  | | Unstandardized | | | Standard Error | | | | t | | | P | | |  |
| H₀ |  | (Intercept) |  | 47.835 | | | 0.094 | | | | 507.218 | | |  | < .001 |  | |
| H₁ |  | (Intercept) |  | 48.022 | | | 0.111 | | | | 433.578 | | |  | < .001 |  | |
|  |  | Sex (M) |  | -0.581 | | | 0.195 | | | | -2.977 | | |  | 0.004 |  | |
|  | | | | | | | | | | | | | | | | | |
|  | | | | | | | | | | | | | | | | | |

Table S3. Analysis of Variance (ANOVA) and Coefficient Tables for the Best Subset Multivariate Regression Model of ***Myotis bechsteinii***.

| ANOVA | | | | | | | | | | | | | | | | | | |  |
| --- | --- | --- | --- | --- | --- | --- | --- | --- | --- | --- | --- | --- | --- | --- | --- | --- | --- | --- | --- |
| Model | |  | | | Sum of Squares | | | | df | | Mean Square | | F | | | P | |  |  |
| H₁ |  | Regression | |  | 4.258 | |  | | 1 |  | 4.258 |  | 4.724 | |  | 0.038 | |  |  |
|  |  | Residual | |  | 27.042 | |  | | 30 |  | 0.901 |  |  | |  |  |  |  |  |
|  |  | Total | |  | 31.300 | |  | | 31 |  |  |  |  | |  |  |  |  |  |
|  | | | | | | | | | | | | | | | | | | |  |
|  | | | | | | | | | | | | | | | | | | |  |
| Coefficients | | | | | | | | | | | | | | | | | | | |
| Model | |  | | Unstandardized | | | | Standard Error | | | | t | | | | P | |  |  |
| H₀ |  | (Intercept) |  | 41.575 | | | | 0.178 | | | |  | | 234.054 | |  | < .001 |  | |
| H₁ |  | (Intercept) |  | 41.918 | | | | 0.230 | | | |  | | 182.038 | |  | < .001 |  | |
|  |  | Sex (M) |  | -0.731 | | | | 0.336 | | | |  | | -2.173 | |  | 0.038 |  | |
|  |  |  |  |  | |  | |  | | |  |  | |  | |  |  |  | |
|  | | | | | | | | | | | | | | | | | | | |

Table S4. Analysis of Variance (ANOVA) and Coefficient Tables for the Best Subset Multivariate Regression Model of ***Myotis capaccinii***. Standardized coefficients are only shown for continuous predictors

| ANOVA | | | | | | | |
| --- | --- | --- | --- | --- | --- | --- | --- |
| Model | |  | Sum of Squares | df | Mean Square | F | P |
| H₁ |  | Regression | 13.026 | 2 | 6.513 | 7.318 | 0.001 |
|  |  | Residual | 52.512 | 59 | 0.890 |  |  |
|  |  | Total | 65.539 | 61 |  |  |  |
|  | | | | | | | |
|  | | | | | | | |
| Coefficients | | | | | | | |
| Model | |  | Unstandardized | Standard Error | Standardized | t | P |
| H₀ |  | (Intercept) | 41.666 | 0.132 |  | 316.515 | < .001 |
| H₁ |  | (Intercept) | 41.609 | 0.231 |  | 179.806 | < .001 |
|  |  | Sex (M) | -1.039 | 0.283 |  | -3.676 | < .001 |
|  |  | Altitude (m) | 0.001 | 7.512×10^-4^ | 0.185 | 1.575 | 0.121 |
|  | | | | | | | |

Table S5. Analysis of Variance (ANOVA) and Coefficient Tables for the Best Subset Multivariate Regression Model of ***Myotis crypticus***. Standardized coefficients are only shown for continuous predictors.

| ANOVA | | | | | | | |
| --- | --- | --- | --- | --- | --- | --- | --- |
| Model | |  | Sum of Squares | df | Mean Square | F | P |
| H₁ |  | Regression | 1.672 | 3 | 0.557 | 0.539 | 0.657 |
|  |  | Residual | 122.028 | 118 | 1.034 |  |  |
|  |  | Total | 123.700 | 121 |  |  |  |
|  | | | | | | | |
|  | | | | | | | |
| Coefficients | | | | | | | |
| Model | |  | Unstandardized | Standard Error | Standardized | t | P |
| H₀ |  | (Intercept) | 38.741 | 0.092 |  | 423.212 | < .001 |
| H₁ |  | (Intercept) | 22.766 | 32.709 |  | 0.696 | 0.488 |
|  |  | Sex (M) | 0.104 | 0.198 |  | 0.524 | 0.601 |
|  |  | Year | 0.008 | 0.016 | 0.048 | 0.496 | 0.621 |
|  |  | Altitude (m) | -2.531×10^-4^ | 2.909×10^-4^ | -0.084 | -0.870 | 0.386 |
|  | | | | | | | |

Table S6. Analysis of Variance (ANOVA) and Coefficient Tables for the Best Subset Multivariate Regression Model of ***Myotis daubentonii***. Standardized coefficients are only shown for continuous predictors.

| ANOVA | | | | | | | | | | | | | |
| --- | --- | --- | --- | --- | --- | --- | --- | --- | --- | --- | --- | --- | --- |
| Model | |  | | Sum of Squares | | df | | Mean Square | | F | | P | |
| H₁ |  | Regression |  | 124.971 |  | 2 |  | 62.485 |  | 68.138 |  | < .001 |  |
|  |  | Residual |  | 564.895 |  | 616 |  | 0.917 |  |  |  |  |  |
|  |  | Total |  | 689.866 |  | 618 |  |  |  |  |  |  |  |
|  | | | | | | | | | | | | | |

| Coefficients | | | | | | | | | | | | | |
| --- | --- | --- | --- | --- | --- | --- | --- | --- | --- | --- | --- | --- | --- |
| Model | |  | | Unstandardized | | Standard Error | | Standardized | | t | | P | |
| H₀ |  | (Intercept) |  | 37.148 |  | 0.042 |  |  |  | 874.775 |  | < .001 |  |
| H₁ |  | (Intercept) |  | -14.738 |  | 8.262 |  |  |  | -1.784 |  | 0.075 |  |
|  |  | Year |  | 0.026 |  | 0.004 |  | 0.231 |  | 6.335 |  | < .001 |  |
|  |  | Sex (M) |  | -0.799 |  | 0.078 |  |  |  | -10.195 |  | < .001 |  |
|  | | | | | | | | | | | | | |

Table S7. Analysis of Variance (ANOVA) and Coefficient Tables for the Best Subset Multivariate Regression Model of ***Myotis emarginatus***.

| ANOVA | | | | | | | |
| --- | --- | --- | --- | --- | --- | --- | --- |
| Model | |  | Sum of Squares | Df | Mean Square | F | P |
| H₁ |  | Regression | 513.298 | 1 | 513.298 | 541.776 | < .001 |
|  |  | Residual | 1.229.774 | 1298 | 0.947 |  |  |
|  |  | Total | 1.743.072 | 1299 |  |  |  |
|  | | | | | | | |
|  | | | | | | | |
| Coefficients | | | | | | | |
| Model | |  | Unstandardized | Standard Error |  | t | P |
| H₀ |  | (Intercept) | 38.803 | 0.032 |  | 1.207.765 | < .001 |
| H₁ |  | (Intercept) | 40.308 | 0.070 |  | 575.297 | < .001 |
|  |  | Sex (M) | -1.767 | 0.076 |  | -23.276 | < .001 |
|  | | | | | | | |

Table S8. Analysis of Variance (ANOVA) and Coefficient Tables for the Best Subset Multivariate Regression Model of ***Myotis mystacinus***. Standardized coefficients are only shown for continuous predictors.

| ANOVA | | | | | | | |
| --- | --- | --- | --- | --- | --- | --- | --- |
| Model | |  | Sum of Squares | df | Mean Square | F | P |
| H₁ | | Regression | 16.502 | 2 | 8.251 | 4.026 | 0.019 |
|  | | Residual | 463.140 | 226 | 2.049 |  |  |
|  | | Total | 479.642 | 228 |  |  |  |
|  | | | | | | | |
|  | | | | | | | |
| Coefficients | | | | | | | |
| Model | |  | Unstandardized | Standard Error | Standardized | t | P |
| H₀ |  | (Intercept) | 34.290 | 0.096 |  | 357.758 | < .001 |
| H₁ |  | (Intercept) | 21.662 | 5.738 |  | 3.775 | < .001 |
|  |  | Sex (M) | -0.440 | 0.223 |  | -1.974 | 0.050 |
|  |  | Latitude | 2.801×10^-6^ | 1.243×10^-6^ | 0.148 | 2.253 | 0.025 |
|  |  |  |  |  |  |  |  |

Table S9. Analysis of Variance (ANOVA) and Coefficient Tables for the Best Subset Multivariate Regression Model of ***Plecotus auritus***. Standardized coefficients are only shown for continuous predictors.

| ANOVA | | | | | | | |
| --- | --- | --- | --- | --- | --- | --- | --- |
| Model | |  | Sum of Squares | df | Mean Square | F | P |
| H₁ |  | Regression | 41.556 | 3 | 13.852 | 12.650 | < .001 |
|  |  | Residual | 196.008 | 179 | 1.095 |  |  |
|  |  | Total | 237.564 | 182 |  |  |  |
|  | | | | | | | |
|  |  |  |  |  |  |  |  |
| Coefficients | | | | | | | |
| Model | |  | Unstandardized | Standard Error | Standardized | t | P |
| H₀ |  | (Intercept) | 39.188 | 0.084 |  | 464.007 | < .001 |
| H₁ |  | (Intercept) | 24.692 | 3.748 |  | 6.588 | < .001 |
|  |  | Altitude (m) | 0.001 | 6.178×10^-4^ | 0.117 | 1.702 | 0.090 |
|  |  | Latitude | 2.927×10^-6^ | 7.779×10^-7^ | 0.261 | 3.763 | < .001 |
|  |  | Sex (M) | -0.800 | 0.158 |  | -5.065 | < .001 |
|  | | | | | | | |

Table S10. Analysis of Variance (ANOVA) and Coefficient Tables for the Best Subset Multivariate Regression Model of ***Barbastella barbastellus***. Standardized coefficients are only shown for continuous predictors.

| ANOVA | | | | | | | |  |
| --- | --- | --- | --- | --- | --- | --- | --- | --- |
| Model | |  | Sum of Squares | df | Mean Square | F | P | |
| H₁ |  | Regression | 104.822 | 2 | 52.411 | 50.929 | < .001 | |
|  |  | Residual | 251.102 | 244 | 1.029 |  |  | |
|  |  | Total | 355.924 | 246 |  |  |  | |
|  | | | | | | | |  |
|  | | | | | | | |  |
| Coefficients | | | | | | | |  |
| Model | |  | Unstandardized | Standard Error | Standardized | t | P | |
| H₀ |  | (Intercept) | 39.857 | 0.077 |  | 520.770 | < .001 | |
| H₁ |  | (Intercept) | 41.448 | 0.218 |  | 189.724 | < .001 | |
|  |  | Sex (M) | -1.124 | 0.156 |  | -7.219 | < .001 | |
|  |  | Altitude (m) | -0.001 | 1.910×10^-4^ | -0.347 | -6.421 | < .001 | |
|  | | | | | | | |  |

Table S11. Analysis of Variance (ANOVA) and Coefficient Tables for the Best Subset Multivariate Regression Model of ***Nyctalus leisleri***. Standardized coefficients are only shown for continuous predictors.

| ANOVA | | | | | | | |  |
| --- | --- | --- | --- | --- | --- | --- | --- | --- |
| Model | |  | Sum of Squares | df | Mean Square | F | P | |
| H₁ |  | Regression | 30.371 | 2 | 15.186 | 13.407 | < .001 | |
|  |  | Residual | 223.130 | 197 | 1.133 |  |  | |
|  |  | Total | 253.501 | 199 |  |  |  | |
|  | | | | | | | |  |
|  | | | | | | | |  |
| Coefficients | | | | | | | |  |
| Model | |  | Unstandardized | Standard Error | Standardized | t | P | |
| H₀ |  | (Intercept) | 43.355 | 0.080 |  | 543.240 | < .001 | |
| H₁ |  | (Intercept) | -27.700 | 25.190 |  | -1.100 | 0.273 | |
|  |  | Year | 0.036 | 0.013 | 0.192 | 2.845 | 0.005 | |
|  |  | Sex (M) | -0.861 | 0.184 |  | -4.692 | < .001 | |
|  | | | | | | | |  |

Table S12. Analysis of Variance (ANOVA) and Coefficient Tables for the Best Subset Multivariate Regression Model of ***Hypsugo savii***.

| ANOVA | | | | | | | |
| --- | --- | --- | --- | --- | --- | --- | --- |
| Model | |  | Sum of Squares | df | Mean Square | F | P |
| H₁ |  | Regression | 141.903 | 1 | 141.903 | 125.463 | < .001 |
|  |  | Residual | 421.877 | 373 | 1.131 |  |  |
|  |  | Total | 563.780 | 374 |  |  |  |
|  | | | | | | | |
|  | | | | | | | |
| Coefficients | | | | | | | |
| Model | |  | Unstandardized | Standard Error |  | t | P |
| H₀ |  | (Intercept) | 33.496 | 0.063 |  | 528.312 | < .001 |
| H₁ |  | (Intercept) | 34.119 | 0.078 |  | 436.365 | < .001 |
|  |  | Sex (M) | -1.230 | 0.110 |  | -11.201 | < .001 |
|  | | | | | | | |

Table S13. Analysis of Variance (ANOVA) and Coefficient Tables for the Best Subset Multivariate Regression Model of ***Pipistrellus kuhlii***. Standardized coefficients are only shown for continuous predictors.

| ANOVA | | | | | | | |
| --- | --- | --- | --- | --- | --- | --- | --- |
| Model | |  | Sum of Squares | df | Mean Square | F | P |
| H₁ |  | Regression | 31.410 | 2 | 15.705 | 16.856 | < .001 |
|  |  | Residual | 87.581 | 94 | 0.932 |  |  |
|  |  | Total | 118.991 | 96 |  |  |  |
|  | | | | | | | |
|  | | | | | | | |
|  |  |  |  |  |  |  |  |
| Coefficients | | | | | | | |
| Model | |  | Unstandardized | Standard Error | Standardized | t | P |
| H₀ |  | (Intercept) | 33.768 | 0.113 |  | 298.724 | < .001 |
| H₁ |  | (Intercept) | 33.139 | 0.242 |  | 136.705 | < .001 |
|  |  | Altitude (m) | 0.001 | 2.302×10^-4^ | 0.390 | 4.391 | < .001 |
|  |  | Sex (M) | -0.686 | 0.204 |  | -3.365 | 0.001 |
|  | | | | | | | |

Table S14. Analysis of Variance (ANOVA) and Coefficient Tables for the Best Subset Multivariate Regression Model of ***Pipistrellus pipistrellus***.

| ANOVA | | | | | | | |
| --- | --- | --- | --- | --- | --- | --- | --- |
| Model | |  | Sum of Squares | df | Mean Square | F | P |
| H₁ |  | Regression | 30.125 | 1 | 30.125 | 47.769 | < .001 |
|  |  | Residual | 95.227 | 151 | 0.631 |  |  |
|  |  | Total | 125.352 | 152 |  |  |  |
|  | | | | | | | |
|  | | | | | | | |
| Coefficients | | | | | | | |
| Model | |  | Unstandardized | Standard Error |  | t | P |
| H₀ |  | (Intercept) | 31.383 | 0.073 |  | 427.464 | < .001 |
| H₁ |  | (Intercept) | 31.796 | 0.088 |  | 362.568 | < .001 |
|  |  | Sex (M) | -0.890 | 0.129 |  | -6.911 | < .001 |
|  | | | | | | | |

Table S15. Analysis of Variance (ANOVA) and Coefficient Tables for the Best Subset Multivariate Regression Model of ***Pipistrellus pygmaeus***. Standardized coefficients are only shown for continuous predictors.

| ANOVA | | | | | | | | |
| --- | --- | --- | --- | --- | --- | --- | --- | --- |
| Model | |  | Sum of Squares | Df | Mean Square | F | P |  |
| H₁ |  | Regression | 21.761 | 3 | 7.254 | 12.428 | < .001 |  |
|  |  | Residual | 13.424 | 23 | 0.584 |  |  |  |
|  |  | Total | 35.185 | 26 |  |  |  |  |
|  | | | | | | | | |
|  | | | | | | | | |
| Coefficients | | | | | | | | |
| Model | |  | Unstandardized | Standard Error | Standardizedᵃ | t | P |  |
| H₀ |  | (Intercept) | 31.059 | 0.224 |  | 138.733 | < .001 |  |
| H₁ |  | (Intercept) | -83.060 | 41.888 |  | -1.983 | 0.059 |  |
|  |  | Year | 0.056 | 0.020 | 0.387 | 2.721 | 0.012 |  |
|  |  | Altitude (m) | 0.002 | 0.002 | 0.220 | 1.300 | 0.207 |  |
|  |  | Sex (M) | -1.008 | 0.427 |  | -2.360 | 0.027 |  |
|  | | | | | | | | |

Table S16. Analysis of Variance (ANOVA) and Coefficient Tables for the Best Subset Multivariate Regression Model of ***Miniopterus schreibersii***. Standardized coefficients are only shown for continuous predictors.

| ANOVA | | | | | | | |
| --- | --- | --- | --- | --- | --- | --- | --- |
| Model | |  | Sum of Squares | Df | Mean Square | F | P |
| H₁ |  | Regression | 46.632 | 3 | 15.544 | 27.834 | < .001 |
|  |  | Residual | 349.586 | 626 | 0.558 |  |  |
|  |  | Total | 396.217 | 629 |  |  |  |
|  | | | | | | | |
|  | | | | | | | |
| Coefficients | | | | | | | |
| Model | |  | Unstandardized | Standard Error | Standardized | t | P |
| H₀ |  | (Intercept) | 45.961 | 0.032 |  | 1.453.515 | < .001 |
| H₁ |  | (Intercept) | 22.483 | 15.357 |  | 1.464 | 0.144 |
|  |  | Year | 0.008 | 0.008 | 0.042 | 1.100 | 0.272 |
|  |  | Latitude | 1.396×10^-6^ | 1.538×10^-7^ | 0.358 | 9.071 | < .001 |
|  |  | Sex (M) | -0.213 | 0.062 |  | -3.423 | < .001 |
|  | | | | | | | |
