# Supplementary material for: Climate is changing, are European bats too? A multispecies analysis of trends in body size
Source: Ecol Evol. 2024 Feb 7;14(2):e10872. doi: 10.1002/ece3.10872 (PMC10850807; doi:10.1002/ece3.10872)
Supplement: Supplementary file 3 — Table S17 [file ECE3-14-e10872-s002.docx]

Table S 17. Subset models of Multiple Linear Regression with Categorical Predictors generated for forearm length (mm) in 15 bat species recorded in Italy. Lat = Latitude (expressed in m, WGS84 coordinate system); Alt = Altitude in m above sea level; P is the overall model’s level of significance. Sex was entered as a categorical predictor.

| *Rhinolophus euryale* |  |  |  |  |
| --- | --- | --- | --- | --- |
| Model | Adj R^2^ | P | N significant terms | Significant terms |
| ['Year'] | - 0.009 | 0.911 | 0 | 0 |
| ['Alt'] | 0.012 | 0.126 | 0 | 0 |
| ['Sex'] | 0.065 | 0.004 | 1 | Sex |
| ['Year', 'Alt'] | 0.003 | 0.308 | 0 | 0 |
| ['Year', 'Sex'] | 0.060 | 0.012 | 1 | Sex |
| ['Alt', 'Sex'] | 0.066 | 0.008 | 1 | Sex |
| ['Year', 'Alt', 'Sex'] | 0.059 | 0.021 | 1 | Sex |
| *Myotis bechsteinii* |  |  |  |  |
| Model | Adj R^2^ | P | N significant terms | Significant terms |
| ['Year'] | 0.0033 | 0.162 | 0 | 0 |
| ['Alt'] | - 0.0030 | 0.761 | 0 | 0 |
| ['Sex'] | 0.107 | 0.038 | 1 | Sex |
| ['Year', 'Alt'] | 0.008 | 0.339 | 0 | 0 |
| ['Year', 'Sex'] | 0.111 | 0.069 | 0 | 0 |
| ['Alt', 'Sex'] | 0.085 | 0.106 | 0 | 0 |
| ['Year', 'Alt', 'Sex'] | 0.092 | 0.130 | 0 | 0 |
| *Myotis capaccinii* |  |  |  |  |
| Model | Adj R^2^ | P | N significant terms | Significant terms |
| Model 1: ['Year'] | - 0.013 | 0.654 | 0 | 0 |
| Model 2: ['Alt'] | - 0.001 | 0.339 | 0 | 0 |
| Model 3: ['Sex'] | 0.151 | <0.001 | 1 | Sex |
| Model 4: ['Year', 'Alt'] | - 0.0017 | 0.622 | 0 | 0 |
| Model 5: ['Year', 'Sex'] | 0.137 | 0.005 | 1 | Sex |
| Model 6: ['Alt', 'Sex'] | 0.172 | 0.001 | 1 | Sex |
| Model 7: ['Year', 'Alt', 'Sex'] | 0.161 | 0.004 | 1 | Sex |
| *Myotis daubentonii* |  |  |  |  |
| Model | Adj R^2^ | *P* | *N significant terms* | *Significant terms* |
| ['Lat'] | - 0.002 | 0.793 | 0 | 0 |
| ['Alt'] | 0.003 | 0.075 | 0 | 0 |
| ['Year', 'Lat'] | 0.054 | <0.001 | 2 | Year, Lat |
| ['Year'] | 0.041 | <0.001 | 1 | Year, Lat |
| ['Lat', 'Alt'] | 0.013 | 0.007 | 2 | Lat, Alt |
| ['Year', 'Lat', 'Alt'] | 0.073 | <0.001 | 2 | Year, Alt |
| ['Year', 'Alt'] | 0.072 | <0.001 | 2 | Year, Alt |
| ['Alt', 'Sex'] | 0.138 | <0.001 | 2 | Year, Alt |
| ['Sex'] | 0.126 | <0.001 | 1 | Sex |
| ['Lat', 'Sex'] | 0.138 | <0.001 | 2 | Lat, Sex |
| ['Lat', 'Alt', 'Sex'] | 0.137 | <0.001 | 1 | Sex |
| ['Year', 'Lat', 'Sex'] | 0.177 | <0.001 | 2 | Sex, Year |
| ['Year', 'Alt', 'Sex'] | 0.177 | <0.001 | 2 | Sex, Year |
| ['Year', 'Sex'] | 0.178 | <0.001 | 2 | Sex, Year |
| ['Year', 'Lat', 'Alt', 'Sex'] | 0.176 | <0.001 | 2 | Sex, Year |
| *Myotis emarginatus* |  |  |  |  |
| Model | Adj R^2^ | P | N significant terms | Significant terms |
| ['Lat'] | 0.014 | <0.001 | 1 | Lat |
| ['Alt'] | 0.158 | <0.001 | 1 | Alt |
| ['Year', 'Lat'] | 0.013 | <0.001 | 1 | Lat |
| ['Year'] | 0.000 | 0.517 | 0 | 0 |
| ['Lat', 'Alt'] | 0.157 | <0.001 | 1 | Alt |
| ['Year', 'Lat', 'Alt'] | 0.159 | <0.001 | 1 | Alt |
| ['Year', 'Alt'] | 0.161 | <0.001 | 1 | Alt |
| ['Alt', 'Sex'] | 0.294 | <0.001 | 1 | Sex |
| ['Sex'] | 0.294 | <0.001 | 1 | Sex |
| ['Lat', 'Sex'] | 0.295 | <0.001 | 1 | Sex |
| ['Lat', 'Alt', 'Sex'] | 0.295 | <0.001 | 1 | Sex |
| ['Year', 'Lat', 'Sex'] | 0.294 | <0.001 | 1 | Sex |
| ['Year', 'Alt', 'Sex'] | 0.293 | <0.001 | 1 | Sex |
| ['Year', 'Sex'] | 0.293 | <0.001 | 1 | Sex |
| ['Year', 'Lat', 'Alt', 'Sex'] | 0.294 | <0.001 | 1 | Sex |
| *Myotis mystacinus* |  |  |  |  |
| Model | Adj R^2^ | P | N significant terms | Significant terms |
| ['Lat'] | 0.013 | 0.044 | 1 | Lat |
| ['Alt'] | - 0.004 | 0.67 | 0 | 0 |
| ['Year', 'Lat'] | 0.010 | 0.121 | 0 | 0 |
| ['Year'] | - 0.003 | 0.54 | 0 | 0 |
| ['Lat', 'Alt'] | 0.012 | 0.096 | 0 | 0 |
| ['Year', 'Lat', 'Alt'] | 0.008 | 1.189 | 0 | 0 |
| ['Year', 'Alt'] | - 0.007 | 0.181 | 0 | 0 |
| ['Alt', 'Sex'] | 0.005 | 0.204 | 0 | 0 |
| ['Sex'] | 0.008 | 0.089 | 0 | 0 |
| ['Lat', 'Sex'] | 0.026 | 0.019 | 2 | Sex, Lat |
| ['Lat', 'Alt', 'Sex'] | 0.026 | 0.031 | 2 | Sex, Lat |
| ['Year', 'Lat', 'Sex'] | 0.022 | 0.046 | 1 | Lat |
| ['Year', 'Alt', 'Sex'] | 0.002 | 0.333 | 0 | 0 |
| ['Year', 'Sex'] | 0.005 | 0.201 | 0 | 0 |
| ['Year', 'Lat', 'Alt', 'Sex'] | 0.021 | ns | 0 | 0 |
| *Plecotus auritus* |  |  |  |  |
| Model | Adj R^2^ | P | N significant terms | Significant terms |
| ['Lat'] | 0.064 | <0.001 | 1 | Lat |
| ['Alt'] | 0.011 | 0.078 | 0 | 0 |
| ['Year', 'Lat'] | 0.035 | 0.014 | 1 | Lat |
| ['Year'] | 0.000 | 0.971 | 0 | 0 |
| ['Lat', 'Alt'] | 0.058 | 0.002 | 2 | Lat, Alt |
| ['Year', 'Lat', 'Alt'] | 0.053 | 0.005 | 2 | Lat, Alt |
| ['Year', 'Alt'] | 0.006 | 0.213 | 0 | 0 |
| ['Alt', 'Sex'] | 0.109 | <0.001 | 1 | Sex |
| ['Sex'] | 0.105 | <0.001 | 1 | Sex |
| ['Lat', 'Sex'] | 0.173 | <0.001 | 2 | Lat, Sex |
| ['Lat', 'Alt', 'Sex'] | 0.183 | <0.001 | 2 | Lat, Sex |
| ['Year', 'Lat', 'Sex'] | 0.171 | <0.001 | 2 | Lat, Sex |
| ['Year', 'Alt', 'Sex'] | 0.110 | <0.001 | 1 | Sex |
| ['Year', 'Sex'] | 0.106 | <0.001 | 1 | Sex |
| ['Year', 'Lat', 'Alt', 'Sex'] | 0.181 | <0.001 |  | Lat, Sex |
| *Barbastella barbastellus* |  |  |  |  |
| Model | Adj R^2^ | P | N significant terms | Significant terms |
| ['Year'] | -0.02 | 0.491 | 0 | 0 |
| ['Alt'] | 0.14 | <0.001 | 1 | Alt |
| ['Sex'] | 0.181 | <0.001 | 1 | Sex |
| ['Year', 'Alt'] | 0.145 | <0.001 | 1 | Alt |
| ['Year', 'Sex'] | 0.186 | <0.001 | 1 | Sex |
| ['Alt'. 'Sex'] | 0.290 | <0.001 | 2 | Sex. Alt |
| ['Year', 'Alt', 'Sex'] | 0.289 | <0.001 | 2 | Sex. Alt |
| *Nyctalus leisleri* |  |  |  |  |
| Model | Adj R^2^ | P | N significant terms | Significant terms |
| ['Lat'] | 0.002 | 0.255 | 0 | none |
| ['Alt'] | 0.009 | 0.09 | 0 | none |
| ['Year', 'Lat'] | 0.012 | 0.117 | 0 | none |
| ['Year'] | 0.017 | 0.039 | 1 | Year |
| ['Lat', 'Alt'] | 0.023 | 0.036 | 2 | Lat, Alt |
| ['Year', 'Lat', 'Alt'] | 0.030 | 0.029 | 1 | Alt |
| ['Year', 'Alt'] | 0.033 | 0.014 | 2 | Year, Alt |
| ['Alt', 'Sex'] | 0.075 | <0.001 | 1 | Sex |
| ['Sex'] | 0.079 | <0.001 | 1 | Sex |
| ['Lat', 'Sex'] | 0.101 | <0.001 | 2 | Lat, Sex |
| ['Lat', 'Alt', 'Sex'] | 0.106 | <0.001 | 2 | Lat, Sex |
| ['Year', 'Lat', 'Sex'] | 0.110 | <0.001 | 1 | Sex |
| ['Year', 'Alt', 'Sex'] | 0.110 | <0.001 | 2 | Sex, Year |
| ['Year', 'Sex'] | 0.111 | <0.001 | 2 | Year, Sex |
| ['Year', 'Lat', 'Alt', 'Sex'] | 0.112 | <0.001 | 1 | Sex |
| *Hypsugo savii* |  |  |  |  |
| Model | Adj R^2^ | P | N significant terms | Significant terms |
| ['Year'] | 0*.*049 | P<0.001 | 1 | year |
| ['Alt'] | 0*.*095 | P<0.001 | 1 | Alt |
| ['Sex'] | 0*.*250 | P<0.001 | 1 | sex |
| ['Year', 'Alt'] | 0*.*094 | P<0.001 | 1 | Alt |
| ['Year', 'Sex'] | 0*.*248 | P<0.001 | 1 | sex |
| ['Alt', 'Sex'] | 0*.*248 | P<0.001 | 1 | sex |
| ['Year', 'Alt', 'Sex'] | 0*.*247 | P<0.001 | 1 | sex |
| *Pipistrellus kuhlii* |  |  |  |  |
| Model | Adj R^2^ | P | N significant terms | Significant terms |
| ['Year'] | 0.013 | 0.135 | 0 | 0 |
| ['Alt'] | 0.167 | <0.001 | 1 | Alt |
| ['Sex'] | 0.105 | <0.001 | 1 | Sex |
| ['Year', 'Alt'] | 0.158 | <0.001 | 1 | Alt |
| ['Year', 'Sex'] | 0.152 | <0.001 | 2 | Year, Sex |
| ['Alt', 'Sex'] | 0.248 | <0.001 | 2 | Alt. Sex |
| ['Year', 'Alt', 'Sex'] | 0.248 | <0.001 | 2 | Alt, Sex |
| *Pipistrellus pipistrellus* |  |  |  |  |
| Model | Adj R^2^ | P | N significant terms | Significant terms |
| ['Lat'] | 0*.*018 | 0*.*099 | 0 | 0 |
| ['Alt'] | 0*.*002 | 0*.*253 | 0 | 0 |
| ['Year', 'Lat'] | 0*.*024 | 0*.*059 | 0 | 0 |
| ['Year'] | - 0*.*006 | 0*.*85 | 0 | 0 |
| ['Lat', 'Alt'] | 0*.*007 | 0*.*223 | 0 | 0 |
| ['Year', 'Lat', 'Alt'] | 0*.*002 | 0*.*358 | 0 | 0 |
| ['Year', 'Alt'] | - 0.004 | 0*.*505 | 0 | 0 |
| ['Alt', 'Sex'] | 0*.*230 | <0.001 | 1 | sex |
| ['Sex'] | 0*.*235 | <0.001 | 1 | sex |
| ['Lat', 'Sex'] | 0*.*231 | <0.001 | 1 | sex |
| ['Lat', 'Alt', 'Sex'] | 0*.*226 | <0.001 | 1 | sex |
| ['Year', 'Lat', 'Sex'] | 0*.*227 | <0.001 | 1 | sex |
| ['Year', 'Alt', 'Sex'] | 0*.*225 | <0.001 | 1 | sex |
| ['Year', 'Sex'] | 0*.*230 | <0.001 | 1 | sex |
| ['Year', 'Lat', 'Alt', 'Sex'] | 0*.*222 | <0.001 | 1 | sex |
| *Pipistrellus pygmaeus* |  |  |  |  |
| Model | Adj R^2^ | P | N significant terms | significant terms |
| ['Year'] | 0.27 | 0.003 | 1 | Year |
| ['Alt'] | 0.215 | 0.009 | 1 | Alt |
| ['Sex'] | 0.468 | <0.001 | 1 | Sex |
| ['Year', 'Alt'] | 0.487 | <0.001 | 2 | Year, Alt |
| ['Year', 'Sex'] | 0.556 | <0.001 | 2 | Year, Sex |
| ['Alt', 'Sex'] | 0.454 | <0.001 | 1 | Sex |
| ['Year', 'Alt', 'Sex'] | 0.569 | <0.001 | 2 | Sex, Year |
| *Miniopterus schreibersii* |  |  |  |  |
| Model | Adj R^2^ | P | N significant terms | Significant terms |
| ['Lat'] | 0.099 | <0.001 | 1 | Lat |
| ['Alt'] | 0.002 | 0.130 | 0 | 0 |
| ['Year', 'Lat'] | 0.098 | <0.001 | 1 | Lat |
| ['Year'] | - 0.001 | 0.631 | 0 | 0 |
| ['Lat', 'Alt'] | 0.098 | <0.001 | 1 | Lat |
| ['Year', 'Lat', 'Alt'] | 0.098 | <0.001 | 1 | Lat |
| ['Year', 'Alt'] | 0.001 | 0.261 | 0 | 0 |
| ['Alt', 'Sex'] | 0.003 | 0.139 | 0 | 0 |
| ['Sex'] | 0.000 | 0.343 | 0 | 0 |
| ['Lat', 'Sex'] | 0.108 | <0.001 | 2 | Lat, Sex |
| ['Lat', 'Alt', 'Sex'] | 0.109 | <0.001 | 2 | Lat, Sex |
| ['Year', 'Lat', 'Sex'] | 0.113 | <0.001 | 2 | Lat, Sex |
| ['Year', 'Alt', 'Sex'] | 0.002 | 0.233 | 0 | 0 |
| ['Year', 'Sex'] | - 0.001 | 0.584 | 0 | 0 |
| ['Year', 'Lat', 'Alt', 'Sex'] | 0.112 | <0.001 | 2 | Lat, Sex |
