# Supplementary material for: Climate is changing, are European bats too? A multispecies analysis of trends in body size
Source: Ecol Evol. 2024 Feb 7;14(2):e10872. doi: 10.1002/ece3.10872 (PMC10850807; doi:10.1002/ece3.10872)
Supplement: Supplementary file 4 — Table S18 [file ECE3-14-e10872-s004.docx]

Table S18. Univariate comparisons of forearm length between sexes for 15 bat species recorded in Italy. Test' statistic: Student’s t-value, * = Mann-Whitney (*U*) statistic.

| Species | df | Test statistic | *P* |
| --- | --- | --- | --- |
| *Rhinolophus euryale* | 113 | 2.98 | 0.004 |
| *Myotis bechsteinii* | 30 | 2.17 | 0.038 |
| *Myotis capaccinii* | 60 | 3.44 | 0.001 |
| *Myotis crypticus* | 121 | -0.41 | 0.685 |
| *Myotis daubentonii* | 617 | 9.51 | <0.001 |
| *Myotis emarginatus* | 1298 | 23.27 | <0.001 |
| *Myotis mystacinus* | - | 5648.5* | 0.04 |
| *Plecotus auritus* | 181 | 4.51 | <0.001 |
| *Barbastella barbastellus* | 245 | 7.430 | <0.001 |
| *Nyctalus leisleri* | 198 | 4.25 | <0.001 |
| *Hypsugo savii* | 373 | 11.20 | <0.001 |
| *Pipistrellus kuhlii* | 96 | 3.53 | <0.001 |
| *Pipistrellus pipistrellus* | 151 | 6.91 | <0.001 |
| *Pipistrellus pygmaeus* | 25 | 4.89 | <0.001 |
| *Miniopterus schreibersii* | 628 | 0.95 | 0.343 |
